# Transition of the automotive industry towards electric vehicle production in the east European integrated periphery

**DOI:** 10.1007/s10663-022-09554-9

**Published:** 2022-11-02

**Authors:** Petr Pavlínek

**Affiliations:** 1 Department of Geography and Geology, University of Nebraska at Omaha, 6001 Dodge Street, Omaha, NE 68182-0199 USA; 2 Charles University, Prague, Czechia

**Keywords:** Automotive industry, Electric vehicles, Eastern Europe, Foreign direct investment, Global value chains, Global production networks, L62, F23

## Abstract

This article analyzes the progress of the transition from the production of vehicles with internal combustion engines to the production of electric vehicles in eastern Europe. The transition is considered in the context of the development of the automotive industry in eastern Europe since the early 1990s and the relative position of the east European integrated periphery in the European automotive industry value chains and production networks. The article argues that foreign firms are driving the transition, while the role of the east European governments and local firms is much less significant. The transition is slower than in western Europe and eastern Europe will continue to produce internal combustion engine vehicles longer. Eastern Europe will continue to rely on its competitive advantage of low production costs, especially low labor costs, to continue to attract foreign direct investment in the automotive industry. The article considers the consequences of the transition for the position of east European countries in automotive value chains, production networks and the division of labor in the European automotive industry.

## Introduction

The European automotive industry has embarked on a transition from the production of vehicles with internal combustion engines (ICEs) to the production of electric vehicles (EVs) [i.e., battery electric vehicles (BEVs) and plug-in-hybrids electric vehicles (PHEVs)], which will lead to the restructuring of the existing automotive industry in Europe. This transition has been necessitated by the adoption of strict CO_2_ emission limits on newly produced vehicles by the European Commission with the goal of decreasing the release of CO_2_ by the transport sector to limit global warming (EC 2019; Pardi 2021; CLEPA 2021; Biresselioglu et al. 2018). The expected adoption of the ‘Fit for 55’ package by the European Union (EU) will effectively ban ICEs in all new cars and vans starting in 2035 (European Council 2022). The automakers would be unable to meet these CO_2_ emission standards with the existing ICE technologies and many view EVs as the only viable alternative (Sigal 2021; McKinsey&Company 2021). However, different automakers have followed different strategies and different technological combinations to meet the emission limits.

The goal of this article is to analyze the impact of this transition in Eastern Europe (EE)^1^ to date in the context of the development of its automotive industry since the early 1990s and its relative position in the European automotive industry value chains and production networks. Theoretically and conceptually, the analysis draws on the global value chains (GVCs)/global production networks (GPNs) perspective (e.g. Gereffi 2018; Kano et al. 2020; Sturgeon et al. 2008; Coe and Yeung 2015; Coe 2021). Although the GVC and GPN perspectives are distinct, they share their focus on the transnational organization of industries in production networks/value chains, power distribution in these networks, the role of various institutions in affecting the configuration and operation of GVCs/GPNs, and the impact of GVCs/GPNs on economic development within the context of the international division of labor (IDL) (e.g., Pavlínek 2018, 2022a). For the purposes of this article the GVC and GPN perspectives are therefore considered as one analytical approach.

I argue that the course of the transition to the production of EVs in EE is strongly affected by the relative position of the EE automotive industry in GVCs/GPNs and the IDL as the integrated periphery of the European automotive industry. I draw on the evolutionary economic geography perspective (e.g. Martin and Sunley 2006; MacKinnon et al. 2019) to contend that this transition is strongly embedded in and constrained by the previous foreign direct investment (FDI) dependent development of the automotive industry in EE (Pavlínek 2017a) and its current integrated periphery position in the European automotive industry production system. This article draws on statistical data about the automotive industry in EE, various automotive industry databases, press reports, specialized automotive industry media, and additional secondary information. It also draws on firm-level interviews previously conducted by the author and members of his research team in Czechia and Slovakia.

The article is organized as follows: First, I briefly summarize the state of the automotive industry in EE. Second, I characterize the relative position of EE in the European automotive industry as the integrated periphery and present its basic features. Third, I explain how the integrated periphery position affects the transition to the production of EVs in EE. Fourth, I discuss the uneven nature of the transition in EE. Fifth, I analyze the development of the battery industry in EE. Finally, I summarize the basic arguments in the conclusion.

## The automotive industry in eastern Europe

A brief overview of the most important features of the automotive industry in EE and its position in the European automotive industry division of labor is a necessary starting point of any analysis of its transition to the production of EVs.

The opening of EE to trade and investment in the early 1990s led to its integration in the European economy, including the rapid development of the export-oriented automotive industry (Van Tulder and Ruigrok 1998; Havas 2000; Pavlínek 2002b, d). Low production costs, market potential, geographic proximity, EU membership or EU preferential trading arrangements, labor surplus in the 1990s and early 2000s, large investment incentives that lowered the set-up sunk costs and thus the investment risk for foreign firms, and other location specific factors attracted foreign automakers and component producers to set up production in EE after 1990 (Pavlínek 2002d, 2008, 2016, 2017a, 2020; Adăscălitei and Guga 2020).

**Fig. 1:**
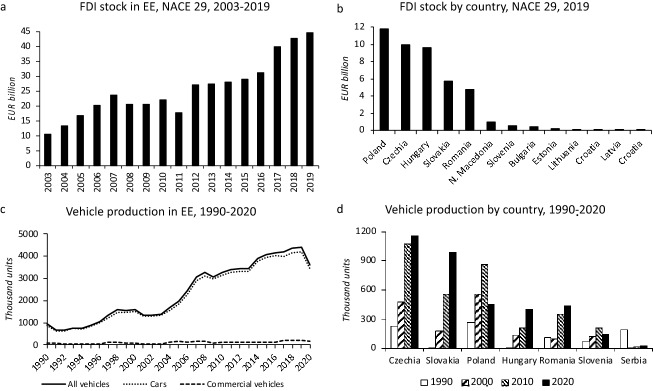
FDI and vehicle production in the EE automotive industry. *Note*: NACE 29 = Manufacture of motor vehicles, trailers, and semi-trailers. *Source*: Author based on data in Eurostat (2022a), OICA (2021), Pavlínek (2002b)

By 2019, the FDI stock in the narrowly defined automotive industry (the manufacture of motor vehicles, trailers and semi-trailers—NACE 29) reached €45 billion in EE (Eurostat 2022a) (Fig. 1a). FDI stock in NACE 29 is highly concentrated in Central Europe (Figs. 1b and 3c). Poland, Czechia, Hungary, and Slovakia together accounted for 83% of the total in 2019, reflecting their geographic, economic, and political location advantages for the automotive industry compared to the rest of EE. As a result of FDI inflows, the production of vehicles and components grew rapidly in EE. Between 1991 and 2019, the output increased 6.6 times from 670 thousand to 4.4 million vehicles (Fig. 1c), accounting for 24.9% of total vehicles produced in the EU in 2019 (OICA 2021). The 2020 production of vehicles decreased by 805 thousand (of which 762 thousand were cars) to 3.6 million in EE because of the COVID-19 pandemic but the EE share of the total EU output increased to 26.2% (OICA 2021). Czechia, Slovakia, and Poland were the largest vehicle producers in 2020 (Fig. 1d). COVID-19 ripple effects, including the shortages of semiconductors, continued to negatively affect the vehicle production in 2021 and 2022. The 2022 production was also negatively affected by the war in Ukraine.^2^

**Fig. 2:**
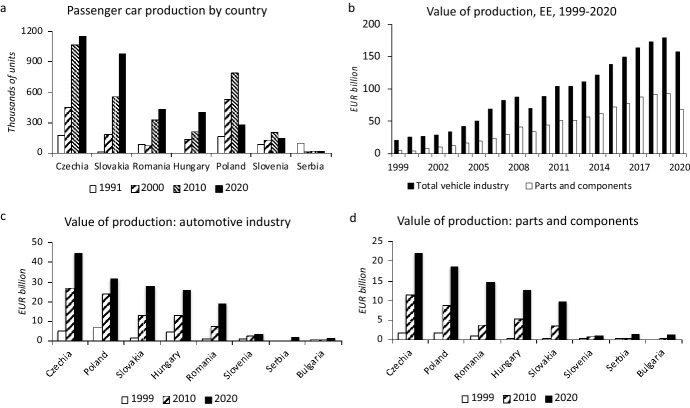
Car production and value of production in the automotive industry of EE. *Source*: Author based on data in Eurostat (2021b, 2022d), OICA (2021), Pavlínek (2002b)

**Fig. 3:**
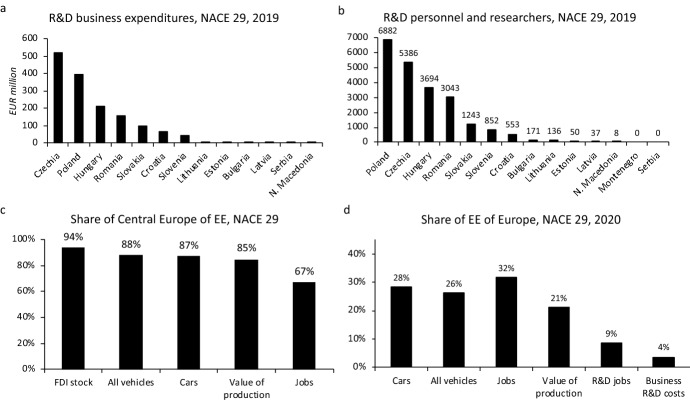
R&D in the automotive industry of EE and regional shares of the automotive industry. *Note*: Data for NACE 29, 2020 data, R&D data for 2019, Europe excludes Russia, Ukraine, Belarus, Turkey. *Source*: Author based on data in Eurostat (2021b, 2022d), OICA (2021)

Prior to the COVID-19 pandemic, the growth was concentrated in the export-oriented production of passenger cars (henceforth cars) which increased almost seven-fold from 863 thousand to 4.2 million between 1991 and 2019 (Fig. 1c). The assembly of cars takes place in Czechia, Slovakia, Romania, Hungary, Poland, Slovenia, and Serbia (Fig. 2a). Central Europe accounted for 87% of the total car production in EE in 2020 (Fig. 3c). Czechia and Slovakia alone accounted for 63%. Compared to cars, the interest of foreign capital in the production of commercial vehicles has been limited in EE. FDI has been concentrated in Poland in the production of light commercial vehicles (LCVs) and heavy trucks. Poland and Czechia are the only two EE countries with a surviving bus production, mainly due to FDI. Czech SOR remains the last significant domestic bus maker in EE because Polish Solaris was sold to Spanish CAF in 2018.

The value of production in the car industry (NACE 29) increased almost eight-fold between 1999 and 2020 (nine-fold between 1999 and 2019) and the value of manufactured parts and components (NACE 29.3) increased 14-fold between 1999 and 2020 (19-fold between 1999 and 2019) (Fig. 2b). The biggest growth was in the 2000s. In the 2010s, the rate of growth slowed, and the value of production doubled between 2000 and 2019. In 2020 the production value of parts and components was higher only by 55% than in 2010 because of the decrease by 26% in 2020 compared to 2019, which was caused by the effects of the COVID-19 pandemic. The distribution of the value of production by country corresponds with the distribution of the car production (Fig. 2c). The largest vehicle producing countries also have the largest production of components (Fig. 2d).

### Limits to growth due to exhausted labor surplus

The declining rates of growth in the 2010s, especially in Central Europe well before the COVID-19 pandemic, reflect the exhaustion of labor surplus by the rapid growth of the automotive industry. It led to labor shortages in the 2010s that pushed wages up, which undermined the rate of profit. For example, in Czechia, which has had the lowest unemployment rate in the EU since 2016, the unemployment rate has been below 3% since 2017 (2.9% in 2017, 2.2% in 2018, 2.0% in 2019, 2.6% in 2020, 2.8% in 2021). Czechia has also consistently had the highest vacancy rate in manufacturing in the EU since 2016 (5.7 in the first quarter of 2022) (Eurostat 2022c). Central Bohemia, which hosts the main production complex of Škoda Auto in Mladá Boleslav, the Toyota factory in Kolín, and many component suppliers, recorded a consistently lower unemployment rate than the national average (2.1% in 2017, 2.0% in 2018, 1.3% in 2019, 1.9% in 2020, and 2.5% in 2021). Similarly, the region of Hradec Králové, which hosts the second Škoda assembly complex at Kvasiny and the Škoda factory at Vrchlabí, recorded a below national average unemployment rate (2.2% in 2017, 2.3% in 2018, 1.6% in 2019, 2.6% in 2020, and 2.3% in 2021). The Moravia-Silesia region, which hosts the Hyundai assembly complex, had the unemployment rate slightly higher than the national average but it was still very low and made it difficult for automotive firms to find the needed workers (4.7% in 2017, 3.7% in 2018, 3.7% in 2019, 3.6% in 2020, and 4.6% in 2021) (CSO 2022). Labor surplus in the Moravia-Silesia region, which was indicated by a high unemployment rate (14.7% in 2003, 14.5% in 2004, 13.9% in 2005) was an important factor in Hyundai’s decision to locate its assembly factory in the Moravia-Silesia region (Pavlínek 2008; CSO 2022). Among 44 foreign-owned automotive firms in Czechia interviewed between 2009 and 2011, 73% reported difficulties in hiring qualified workers despite the economic crisis (e.g., Pavlínek and Ženka 2010; Pavlínek 2015a).

Poland (3.4%) and Hungary (4.1%) had the third and fifth lowest unemployment rate in the EU in 2021 (Eurostat 2022e). Hungary has had the second highest vacancy rate in manufacturing in Central Europe (3.1 in the first quarter of 2022), while Poland’s vacancy rate has been much lower (1.1 in the first quarter of 2022) (Eurostat 2022c). However, the national level data do not reveal large regional differences in labor availability that are accentuated by the clustering of the automotive industry in regional production complexes (e.g., Sturgeon et al. 2008). For instance, the national unemployment rate in Slovakia has been higher than in the rest of Central Europe (6.8% in 2021) and its vacancy rate in manufacturing has been among the lowest in the EU (1.0 in the first quarter of 2022) (Eurostat 2022c, e). Still, the Slovak automotive industry has experienced severe labor shortages in regions targeted by automotive FDI, especially in West Slovakia, which, along with the Bratislava region, hosts the largest share of FDI in the Slovak automotive industry, including four assembly factories and hundreds of component suppliers (e.g. Jacobs 2016; Pavlínek 2016). In 2001, the unemployment rate of West Slovakia (NUTS 2) was 17.5%. It decreased to 4.0% in 2019 and was at 4.7% in 2021 (SSO 2022). The Bratislava region, which hosts a large VW assembly factory complex and many automotive suppliers, had an unemployment rate of 2.6% in 2021 (SSO 2022). The very low unemployment rate translated in severe labor shortages for automotive firms in western Slovakia and became a barrier for the further development of the automotive industry. Interviews with 27 foreign owned automotive industry firms conducted by the author in western Slovakia between 2011 and 2015 revealed that 96% of the interviewed firms had major difficulties hiring qualified workers in Slovakia. Only one supplier argued that it did not face major difficulties, but at the expense of busing workers to its factory from places located up to 100 km away. In 2018, 82% of 61 surveyed automotive suppliers in Slovakia identified the lack of available qualified workers on the job market as a risk factor affecting their future growth prospects, 78% considered the unavailability and low quality of labor a major issue for their company, and 53% (up from 37% in 2016) argued that the lack of skilled labor restricted their ability to win or accept new contracts (PwC 2018).

Similar widespread labor shortages in the automotive industry have been reported from other EE countries, including Hungary (HIPA 2020; Szabo et al. 2022) and Romania (Guga 2019; Adăscăliței and Guga 2020) and are considered the most important barrier to future investment across EE, which also holds for western Europe (WE) (Slačík 2022). Labor shortages have forced automotive firms to increasingly rely on foreign workers and agency employment. More importantly, in line with theories of uneven economic development (e.g., Harvey 2005), some automakers and component suppliers, especially those engaged in labor-intensive production, have been increasingly looking for new potentially more profitable locations with labor surplus and low labor costs for future investments in countries such as Serbia, Moldova, Bosnia and Herzegovina, and North Macedonia (Pavlínek 2018, 2020). PSA and Renault have set up assembly plants in Morocco, with a projected capacity to reach 700 thousand cars by the end of 2022 and Morocco is aiming for 1 million assembled vehicles per year by the mid-2020s (Bolduc 2017; Henry 2020).

### Upgrading and higher value-added functions in the EE automotive industry

FDI in the EE automotive industry has led to the development of a distinct division of labor in the European automotive industry. By investing in EE, foreign firms have mainly pursued cost cutting to increase their profitability and competitiveness (Pavlínek 2020, 2002d). This has translated in the focus on setting up production functions, while higher value-added functions remained concentrated in the home countries of foreign investors (Pavlínek and Ženka 2016; Pavlínek 2016, 2022a). In the 1990s, the focus in EE was on the low value-added labor-intensive assembly operations, often based on cross-border investment in the production of components and car assembly (Pavlínek 1998; Pavlínek and Smith 1998). Over time, however, there has been the gradual upgrading of the production to more sophisticated and capital-intensive automotive production of high-quality cars and components (Pavlínek et al. 2009; Pavlínek and Ženka 2011), in which low labor costs continue to play an important role in keeping production costs under control and thus contributing to the overall competitiveness of finished products and also of lead automotive firms (Boyer and Freyssenet 2002) (Table 1). Foreign assembly firms and many component suppliers are now making cars and components in state-of-the-art factories based on advanced technologies in EE (Layan 2006). There is therefore no doubt about FDI-driven process and product upgrading (Humphrey and Schmitz 2002) in the automotive industry of EE since the 1990s (Layan 2006; Pavlínek et al. 2009; Szalavetz 2019), although not all foreign firms have been successful in EE, as evidenced for example by the failure of Daewoo investments (Pavlínek 2006). Process and product upgrading has also been crucial for the competitiveness and survival of local (domestic) firms (Pavlínek and Ženka 2011). Some surviving or newly established local automotive firms have successfully internationalized (Micek et al. 2021), although many local firms did not survive because the most successful ones were taken over by foreign firms (Pavlínek 2002c), while unsuccessful ones ended in bankruptcy (Pavlínek 2000, 2002a, 2003) and the overall growth of local firms has been much slower than the growth of foreign firms (Pavlínek 2020). These processes have contributed to the overwhelming foreign control of the automotive industry in EE (Table 2).

**Table 1:** Labor cost per employee full-time equivalent in thousand euro (at exchange rate parity) in the European automotive industry (NACE 29) by country in 2019

	Thousands of EUR	Germany = 100
Germany	87.9	100.0
Switzerland	85.5^a^	97.3
Ireland	84.3	95.9
Sweden	78.0	88.7
Belgium	72.6	82.6
Austria	71.5	81.3
France	71.2	81.0
Denmark	69.8	79.4
Norway	67.8	77.1
Netherlands	62.3	70.9
Iceland	61.9^b^	70.4
Italy	61.8	70.3
Britain	54.6^b^	62.1
Finland	48.9	55.6
Spain	47.4	53.9
Czechia	26.0	29.6
Slovakia	25.2	28.7
Portugal	24.4	27.8
Estonia	23.5	26.7
Hungary	23.0	26.2
Greece	21.3	24.2
Poland	19.4	22.1
Latvia	18.5	21.0
Cyprus	17.7	20.1
Lithuania	16.9	19.2
Croatia	15.0	17.1
Romania	14.5	16.5
Turkey	14.0^c^	15.9
Bosnia and Herzegovina	10.4	11.8
Bulgaria	8.6	9.8
North Macedonia	5.0^d^	5.7

^a^2016, ^b^2018, ^c^2014, ^d^2012

*Source*: Eurostat (2022d)

**Table 2:** The index of foreign control in the European automotive industry, 2019

Slovakia	97.9
Hungary	96.3
Romania	94.2
Czechia	93.4
Bulgaria	92.0
Poland	89.7
Spain	85.9
Portugal	84.5
Britain	83.6^b^
Lithuania	83.6
Slovenia	83.3
Austria	80.1
Bosnia and Herzegovina	79.9^a^
Belgium	74.9
Sweden	63.5
Netherlands	58.1^a^
Estonia	57.2
Croatia	54.4^a^
Ireland	49.2
Denmark	44.6
Finland	31.3
Norway	25.1
France	24.1
Italy	23.6
Germany	14.9

The average value of the share of foreign controlled enterprises of five indicators in the manufacture of motor vehicles, trailers, and semi-trailers (NACE_R2): production value, value added at factor cost, gross investment in tangible goods, number of persons employed, and turnover or gross premiums written

^a^2018, ^b^2017

*Source*: Calculated by author from data available in Eurostat (2022b, d)

At the same time, foreign firms have invested disproportionately less in functional upgrading and the development of the higher value-added functions in the automotive industry, including research and development (R&D) in EE (Pavlínek 2004, 2012, 2020; Domański and Gwosdz 2009; Darteyre and Guga 2022) (Fig. 3a, b). Foreign-controlled R&D employment and R&D investment gradually increased in EE as the low cost of the R&D labor force attracted FDI and there are numerous examples of a successful automotive R&D developed by foreign firms in EE (Pavlínek et al. 2009; Pavlínek 2012; Szalavetz 2019; Markiewicz 2019; Guzik et al. 2020). However, important barriers exist, which are related to the organization of corporate R&D in the automotive industry (Pavlínek 2012), as well as the shortages of the qualified R&D labor in EE (Pavlínek 2018; Szalavetz 2022). Consequently, the share of R&D employment and R&D expenditures in the EE automotive industry remains low compared to WE (Tables 3 and 4) (Pavlínek 2022a). While EE accounted for 32% jobs in the EU automotive industry in 2020, its share of R&D jobs was 8.7% and the share of R&D business expenditures was only 3.6% in 2019 (Fig. 3d). The overall weakness of automotive R&D in EE is also illustrated by the very low number of patents compared to WE (Delanote et al. 2022). Although selective functional upgrading in functions other than R&D in foreign subsidiaries has gradually developed (Sass and Szalavetz 2013; Szalavetz 2022), empirical firm-level research has uncovered the weak presence of strategic and high value-added functions in the foreign subsidiaries of automotive firms in the EE automotive industry (Pavlínek and Ženka 2016; Pavlínek 2016), which is closely related to the distribution of functions in the corporate hierarchy (Hymer 1972; Pavlínek 2012).

**Table 3:** The share of business R&D expenditures of the total value of production in the automotive industry (NACE 29) of selected European countries in 2019

	Percent	Germany = 100
Sweden	7.42	105.2
Germany	7.06	100.0
Britain	4.54^a^	64.3
Austria	3.54	50.2
Italy	2.82	40.0
Norway	2.67	37.8
Finland	2.63	37.3
France	2.38^b^	33.8
Malta	1.70	24.1
Belgium	1.37	19.5
Slovenia	1.20	17.0
Poland	1.05	14.9
Czechia	1.01	14.4
Lithuania	0.97	13.7
Netherlands	0.92^c^	13.0
Estonia	0.88	12.4
Ireland	0.83	11.7
Spain	0.79	11.3
Romania	0.76	10.8
Hungary	0.73	10.4
Denmark	0.64	9.1
Latvia	0.51^d^	7.2
Portugal	0.38	5.4
Slovakia	0.33	4.7
Bulgaria	0.23	3.3
Serbia	0.02	0.3

^a^2018, ^b^2017, ^c^2012, ^d^2015. The value for Sweden calculated from the total for NACE 29 and NACE 30 (the NACE 29 data not available)

*Source*: Calculated by author based on data in Eurostat (2021a, 2022d), Statistics Sweden (2021)

**Table 4:** The share of R&D personnel and researchers of total persons employed in the automotive industry (NACE 29) of selected European countries in 2019

	Percent	Germany = 100
Germany	16.06	100.0
Sweden	13.09	81.5
Britain	11.58	72.1
Austria	10.32	64.3
Italy	9.68	60.3
Norway	8.17	50.8
France	7.69	47.8
Netherlands	7.57	47.1
Finland	5.20	32.3
Slovenia	4.90	30.5
Turkey	4.82	30.0
Belgium	4.57	28.5
Spain	4.56	28.4
Hungary	3.56	22.2
Portugal	3.08	19.2
Poland	3.07	19.1
Czechia	2.96	18.4
Ireland	2.51	15.6
Denmark	2.25	14.0
Lithuania	2.21	13.7
Estonia	1.70	10.6
Romania	1.69	10.5
Latvia	1.57	9.8
Slovakia	1.52	9.4
Bulgaria	0.76	4.7
North Macedonia	0.04	0.3

The value for Sweden calculated from the total for NACE 29 and NACE 30 (the NACE 29 data not available)

Calculated by author based on data in Eurostat (2021b, 2022d), Statistics Sweden (2021)

## The integrated periphery of the European automotive industry

The uncritical and simplistic accounts of the development of the automotive industry in EE view it as an unqualified success by emphasizing short-term capital, employment, and production effects of FDI (Jakubiak et al. 2008; Kureková 2012; Kureková Mýtna 2018; Markiewicz 2019). These accounts tend to present the growth of the automotive industry as a success of the national economy by ignoring the fact that it is mainly the result of large FDI inflows and has very little to do with the nature and the level of development of the national economy. At the same time, these accounts either underplay or completely ignore the potential long-term effects of the foreign-capital driven development in the form of newly created dependencies (capital, technological, financial, decision making) and the outflow of value in the form of dividends and profit repatriation (Dischinger et al. 2014a, 2014b) that will affect the ability of EE countries to improve their position in the IDL and close the development gap with the more developed countries of WE (Pavlínek 2022b).

For example, in Slovakia, the government agencies, politicians and the media frequently argue that Slovakia is a global automotive industry “superpower” because it has achieved the highest production of cars per capita in the world (e.g. Sario 2022; ZAP 2022). This simplistic account of the automotive industry in Slovakia based on a single indicator ignores the fact that the automotive industry is almost completely controlled by foreign capital and Slovakia has the highest index of foreign control in the EU at 97.9% in 2019 (Table 2). The share of foreign capital of production value, value added at factor cost and turnover exceeds 99% (Eurostat 2022b, d). All cars are assembled in foreign-owned factories based on foreign technology, work organization and management and R&D (see Pavlínek 2016). The foreign-controlled automotive industry is mostly isolated from the Slovak economy because it has only tenuous linkages with local firms, which diminishes a potential for spillovers from foreign to local firms (Pavlínek 2018). By far the most important production factor Slovakia contributes to the automotive industry is its relatively low-cost labor compared to WE (Table 1). Instead of being the global automotive industry superpower and despite the highest per capita production of cars in the world, an empirical analysis has demonstrated Slovakia’s peripheral position in the European automotive industry production system, which is almost totally controlled from the core areas of the global automotive industry. Other countries of the EE integrated periphery are in a similar highly dependent peripheral position in the automotive GVCs/GPNs (Table 2) (Pavlínek 2022a).

It is, therefore, important to understand the course of the current and future transition to the production of EVs in EE from an evolutionary perspective and in the context of its relative position as the integrated periphery in the European automotive industry GVCs/GPNs (Pavlínek 2018, 2020, 2022a). Since the concept of the integrated periphery has been theoretically and conceptually developed elsewhere (Pavlínek 2018, 2020), its discussion here is limited to a brief summary of basic features applied to the automotive industry of EE. At the general level, Pavlínek (2018: 144) has defined an integrated periphery as “a dynamic area of relatively low-cost (industrial) production that is geographically adjacent to a large market and has been integrated within a core-based macro-regional production network through FDI. In an integrated periphery, production, organization, and strategic functions in a given industry are externally controlled through foreign ownership.” Accordingly, Pavlínek (2018, 2020) has identified the basic features of the integrated periphery of the European automotive industry in EE as follows:

1. Substantially lower labor costs than in the core regions of the European automotive industry (Pavlínek 2022a), such as Germany, France, and Italy, despite a smaller wage gap in 2019 than in the 1990s when wages in EE were about 90% lower than in WE (Table 1).^3^
2. A sizeable labor surplus at the initial stages of growth of the automotive industry, which, however, becomes exhausted over time because of the FDI-driven growth of the automotive industry, leading to labor shortages that undermine the future growth prospects (e.g. PwC 2019; HIPA 2020).
3. The geographic proximity to large and lucrative markets in core regions of WE, especially Germany. It lowers transportation costs of automotive products from integrated peripheries to core areas and vice versa and is further supported by the development of modern transport infrastructure in integrated peripheries, such as divided highways and modernized high-speed railways.
4. The membership in the EU or preferential trading arrangements with the EU in the cases of non-EU countries that provide tariff-free access to EU markets.
5. A high degree of foreign ownership and control over the automotive industry through FDI, which is the highest in the EU. It usually exceeds 90% for the most important automotive industry countries of EE (Table 2).
6. An export-oriented high-volume production focusing on standardized cars and generic automotive components, along with low-volume production of niche-market vehicles (Havas 2000; Pavlínek 2002d; Layan 2006). Typically, more than 90% of assembled vehicles are exported (Pavlínek 2018; WTEx 2021; OEC 2022).
7. A regional specialization based on the spatial division of labor resulting from the strategy of complementary specialization (Kurz and Wittke 1998), in which the integrated periphery has a greater share of low value-added labor-intensive production tasks compared to the automotive industry in WE (Pavlínek 2002d; Jürgens and Krzywdzinski 2009a; Stöllinger 2021; Slačík 2022).
8. The weak presence of high value-added and strategic functions, such as R&D and strategic decision making compared to the extent of production functions in integrated peripheries (Tables 3 and 4, Fig. 3) (Pavlínek 2012, 2016, 2022a; Pavlínek and Ženka 2016; Stöllinger 2021; Slačík 2022; Delanote et al. 2022), resulting in the truncated development of the automotive industry (Pavlínek 2017b).
9. FDI-friendly state policies, large investment incentives, low corporate taxes, and an active state competition over strategic automotive FDI with other countries contributing to the ‘race to the bottom’ in the integrated periphery (Drahokoupil 2008, 2009; Pavlínek 2016).
10. Weak labor unions, more liberal labor codes and more flexible labor practices compared to the automotive industry core countries, especially Germany (Jürgens and Krzywdzinski 2009a, b; Drahokoupil and Myant 2017; Martišková et al. 2021).
11. A weakly developed domestic automotive industry compared to the foreign-controlled automotive sector (Table 2) (Pavlínek 2018, 2020) resulting in the integration of domestic firms into macro-regional GVCs/GPNs at an inferior and subordinate position mainly as low-cost Tier-3 suppliers of niche products and simple parts and components (Pavlínek and Janák 2007; Pavlínek and Žížalová 2016; Pavlínek 2018).

Overall, there is no doubt that the post 1990 development of the automotive industry in EE has been very successful when measured by production volumes, jobs created, capital invested, the contribution to GDP and foreign trade, and other quantitative indicators (Figs. 1 and 2) (e.g., Delanote et al. 2022; Slačík 2022). At the same time, however, the foreign-controlled automotive industry in EE has been articulated into automotive GVCs/GPNs via FDI and trade in a dependent and subordinated position through what the GPN perspective calls the structural mode of strategic coupling between regional assets and the needs of TNCs (Coe and Yeung 2015; Coe 2021). More specifically, it has been mostly articulated as an ‘assembly platform’ that concentrates on production functions and has weakly developed strategic functions (Pavlínek 2016; Pavlínek and Ženka 2016; Stöllinger 2021; Slačík 2022; Delanote et al. 2022). It is also typified by weak linkages of foreign-owned automotive firms with host country economies that translate into weak spillovers from foreign firms to host country economies (Pavlínek and Žížalová 2016; Pavlínek 2018). This situation contributes to a low value capture from the automotive industry compared to the automotive industry in WE and has long-term structural consequences for the EE integrated periphery, especially for its ability to close the development gap, wage levels and the standard of living with more developed WE (Pavlínek 2018, 2020, 2022a, b).

## The integrated periphery and the transition to the production of EVs in eastern Europe

The relative position of the EE integrated periphery in the European automotive industry GVCs/GPNs will influence the course of its transition to the production of EVs. The starting point of my analysis is the assumption of inevitability of the transition away from the production of ICE vehicles, which is based on three points. First, the emission limits imposed on the EU automotive industry by the European Commission (EC 2019; Pardi 2021; CLEPA 2021) cannot be met without shifting the production away from ICE vehicles. Second, feasible technological options for the automotive industry to meet these limits by the deadline specified in the EU regulations are currently limited. Consequently, a consensus has emerged in the automotive industry about meeting the emission limits and regulations by shifting to the production of EVs (BEVs, PHEVs and hybrids) (Jetin 2020; Sigal 2021; McKinsey&Company 2021). Third, the automotive industry trends in the direction of EVs in China (Schwabe 2020a; Yeung 2019) and the United States (Slowik and Lutsey 2018), the largest and third largest (after the EU) automobile markets in the world, generate regulatory and competitive pressures on the European automakers to embrace the EV technology. This pressure applies in foreign markets (especially in China) in the form of state regulation (Yeung 2019; Schwabe 2020a) and the growing competition from local (Chinese and American) carmakers in EVs. It also applies in the EU markets because of the growing competition in EVs from foreign firms, especially the American Tesla, EVs made by foreign firms in China that will be imported to Europe (e.g. the Mini EV made by GM at Wuling), and from Chinese automakers (Sigal 2022b; Manthey 2021a).^4^ At the same time, the transition to the production of EVs is risky and extremely costly for the automotive industry (Dijk et al. 2016; Delanote et al. 2022) and involves many uncertainties for automotive firms and suppliers (CLEPA 2021). The failure of the EU-based automakers to succeed would have serious repercussions not only for the European automotive industry but for the entire European economy (ACEA 2022a).

In terms of the EE automotive industry, several general observations can be made about how its position in GVCs/GPNs and the IDL in the automotive industry will affect its transition to the production of EVs.

### EE is not the center of innovation for electromobility

First, EE, is not and will not be the center of innovation for electromobility. R&D for EVs is mainly conducted in the home countries of assembly firms and large ‘global’ Tier 1 suppliers, which are mostly located in WE, the United States, Japan, and South Korea. With the partial exception of Škoda Auto and Dacia, which is related to their position as Tier Two lead firms (Pavlínek and Janák 2007; Pavlínek 2015b) and few additional examples of R&D developed by assembly firms, such as 400 R&D workers working in technical development at Audi Hungária (Audi 2021), R&D competencies of car makers are very limited or completely absent in EE (Pavlínek 2012). It also applies to the supplier sector despite the selective development of R&D activities by foreign TNCs in EE (Pavlínek et al. 2009; Pavlínek 2012; Guzik et al. 2020), as reflected in the low share of business R&D expenditures of the total value of production and the low share of R&D personnel and researchers of total persons employed in the automotive industry (Tables 3 and 4). This situation is a typical feature of ‘truncated development’, which refers to the absence or low share of high value-added activities, such as R&D functions, strategic planning, and the decision making about major investments, in foreign-owned factories in host regions, and their concentration at home countries of foreign investors, usually at corporate headquarters and corporate R&D centers (Britton 1980, 1981; Hayter 1982; Pavlínek 2017b). The truncated development is strongly pronounced in the EE automotive industry because of the very high degree of foreign ownership and control (Table 2) (Pavlínek 2016; Pavlínek and Ženka 2016), and despite the fact that innovation activities in the automotive industry, including some R&D functions related to electromobility, gradually and selectively spread from core areas to the integrated peripheries of the automotive industry (Friedmann 1967; Pavlínek 2012, 2022a; Pavlínek et al. 2009). For example, Škoda Auto has been developing new R&D competencies in Czechia related to the transition to the production of EVs (Škoda Auto 2021c), although these R&D competencies are much weaker than the ones performed by the main VW’s corporate R&D center in Germany. Despite the gradual and selective development of innovative activities mostly driven by cheaper R&D labor in EE than in WE, the intensity and size of innovation activities will continue to be much stronger in the core areas than in the integrated periphery. The main reason is better conditions for innovation activities in core areas (Isaksen and Trippl 2017; Tödtling and Trippl 2005), which is reflected in higher automotive industry R&D employment and R&D spending in WE compared to EE (Tables 3 and 4).

### A slower pace of transition to the production of EVs than in WE

The second general observation about the transition to the production of EVs in EE is its slower speed than in WE, especially when compared to the core countries of the European automotive industry (Germany, France, Italy). For example, Renault plans to sell 90% BEVs by 2030 but its Romania-based low-cost brand Dacia might reach only 10% BEVs according to the Renault Group’s director of R&D (Randall 2021) and it plans to sell ICEs “for as long as it can” according to Dacia’s CEO (ANE 2022a). While fully dedicated factories for the large-scale production of EVs have been opened in WE (e.g., VW’s factories at Zwickau and Emden, Tesla’s factory near Berlin) or are being planned (e.g., VW’s Trinity factory near Wolfsburg), EE factories have so far employed the strategy of mixed production, in which EVs are assembled along with ICE vehicles in the same factory. This strategy will make it more difficult to achieve scale economies and, therefore, lower production costs of EVs. To make this kind of mixed production viable in the short- and medium-run, EE factories plan to compensate with lower production costs and high labor flexibility. In the long run, however, this strategy is not competitive with the production in fully dedicated EV factories and the production model in which each assembly line is fully dedicated to one platform (Gibbs 2019b). Consequently, the mixed production strategy may become a major disadvantage for the competitive position of EE factories in the future. As of now, there are only two known exceptions to the mixed production strategy in EE. The first is the BMW factory, which is under construction in Debrecen, Hungary, and which should be completed in 2025. The Debrecen factory was originally also planned for the mixed production of models with ICEs and electrified drivetrains (BMW 2018). However, after first delaying the factory construction and the production launch by 3 years, it was decided to fully dedicate the factory to the production of EVs (BMW 2020). The second exception is the Volvo factory announced in 2022 that will be built in Slovakia between 2023 and 2026 (Hampel 2022). These two cases suggest that factories fully dedicated to EVs will eventually be developed in EE to serve the EU markets and will likely exist alongside the factories producing vehicles based on ICEs for non-EU markets.

### Longer production of ICE vehicles and internal combustion engines than in WE

Third, the production of ICE cars and ICEs will continue longer in EE than in WE. In some cases, the production of ICE vehicles and ICEs is being transferred to EE from WE, which might benefit the EE locations in the short and medium run by additional investment, job creation and increased production. For example, VW is transferring the production of the VW Passat from Germany to Slovakia to make space for the production of EVs in Germany (VW 2021) or Stellantis is increasing the production capacity of its engine factory by 50% in Szentgotthárd, Hungary to start the production of new 1.6-L petrol engines in the first half of 2023 (Hungarian Insider 2021). The restructuring of the ICE production in Europe will entail either the closure of ICE factories or their conversion to the production of electric engines or batteries in the high-wage European automotive industry core countries, such as Germany and France. The remaining ICE production will move to countries with lower wages in the integrated periphery, such as EE (Sigal 2022a). The production of ICE cars will continue longer in EE than in WE for several reasons: (1) there are newer, more modern assembly factories than in WE; (2) older technologies continue longer in peripheral locations than in core locations of spatial systems according to the product life cycle model (Vernon 1966); (3) EE has the advantage of lower production costs than in WE (Table 1); (4) EE will continue to produce ICE vehicles for non-EU markets, such as Škoda Auto, which will produce ICE cars for the markets in less developed regions, such as India, Southeast Asia, South America, and Africa (Škoda Auto 2021b); and (5) the transition to the production of EVs in EE will mainly be driven by foreign demand. More than 90% of cars produced in EE are exported and the demand for EVs has been low in EE compared to WE because of higher prices of EVs compared to cars with ICEs and limited subsidies for the purchase of EVs (ACEA 2021c, 2022b). It will make sense for the automakers to continue to make ICE cars close to the market in EE where they also will be sold. For all these reasons, we may assume that the EE integrated periphery will be the last region in the EU to completely shut down the production of ICE vehicles. The production of ICE cars will continue for at least an additional 20 years unless there will be a political decision by the European Commission banning the production and sale of ICE cars sooner. However, relying on the continuing production of ICE cars is a risky strategy for the EE automotive industry, because the delay in the introduction of the large volume production of EVs might undermine its long-term competitiveness. The continuing specialization in the ICE technology, which will rapidly become obsolete, instead of the cutting-edge BEV technology, might result in a long-term disadvantage in the EE automotive industry compared to countries and regions that undergo a rapid transition to the production of EVs.

### The dependence of the EE automotive industry future on foreign TNCs

Fourth, the high degree of foreign control over the EE automotive industry (Table 2) means that the future of the EE automotive industry, including the course of the transition to the production of EVs will be decided abroad by large foreign-owned assembly firms and component suppliers through their corporate decisions about the allocation of production and investment. Flagship foreign investors achieved the ‘corporate capture’ of national and local institutions and resources in EE, which primarily serve the needs of foreign TNCs, often at the expense of local firms and other local needs (Phelps 2000, 2008; Drahokoupil 2008, 2009; Pavlínek 2016). The role of EE governments will be mostly limited to the efforts to influence these corporate decisions via the provision of various investment incentives to attract automotive FDI, especially flagship investors (Pavlínek 2016), including FDI into battery manufacturing (e.g. €267 million in investment incentives to Volvo to build the assembly factory in Košice by Slovakia, €209 million state aid to SK On for the construction of the battery plant in Iváncsa and €108 million awarded to Samsung SDI for the expansion of its battery cell plant in Göd by Hungary, €95 m in aid given to LG Energy Solution to expand the battery plant in Wrocław by Poland, and large investment incentives promised by Czechia for the construction of a battery gigafactory) (Tables 5 and 6). While EE countries are willing to offer large investment incentives to flagship investors, especially assembly firms, large suppliers, and battery manufacturers, they have otherwise followed mostly a wait and see strategy. Consequently, the support of the state for the transition to the production of EVs beyond investment incentives has been limited so far. There has been uneven but mostly weak state support for the building of infrastructure (charging stations) (Transport & Environment 2020; ACEA 2021b; Grzegorczyk 2021; Darteyre and Guga 2022) and uneven state support for the purchase of EVs. For example, as of 2022, Hungary, Romania and Croatia offer generous purchase incentives, smaller incentives are provided in Lithuania, Poland and Slovenia, and no incentives for individuals are in place in Bulgaria, Czechia, Estonia, Latvia, and Slovakia (ACEA 2022b). Poland represents an interesting exception. Its government has actively attempted to break out of the FDI dependency in the transition to EVs by launching the project of the national BEV, the Izera, in 2020. The Izera project strongly depends on foreign technologies and know-how from lead firms such as VW and it is not clear, whether the assembly will be launched in 2024 as planned (Đorđević 2021). In some cases, EE governments have been hostile to EC regulations and the transition to EVs. In Czechia, for example, the Czech Prime Minister Andrej Babiš argued in 2021 “We have repeatedly said that the [EU’s climate] goals must be set in a way not to harm our industry…It must be done reasonably, not based on ideology” (Prague Morning, 2021). Following the 2021 elections, the new prime minister of Czechia Petr Fiala declared on December 19, 2021: “the proposal of the European Commission to ban the production and sales of [new] ICE cars after 2035 is unacceptable for the government of Czechia” (Aktuálně.cz 2021). The new minister of industry and trade of Czechia added: “I think it's nonsense to ban the sale of internal combustion engines.” (Prokeš 2021c). The weak role of the state in the transition to the production of EVs as a mere facilitator (Horner 2017) in EE reinforces the assumption that the future of the EE automotive industry will mainly depend on the corporate strategies of foreign TNCs.

**Table 5:** Battery gigafactories in EE, including announced projects

Company and home country	2021 capacity (GWh)	Start date	Location	Notes
LG Energy Solution, South Korea	45.0	2018, expansion 2022	Wrocław, Poland	The expansion to 65–70 GWh in 2022. Total investment €1.5 billion. 6000 full-time workers planned by the end of 2022. Investment incentives €95 million to expand the plant
SK innovation, South Korea	7.5	2020	Komárom Plant 1, Hungary	The expansion to 23.5 GWh by 2023. Investment €688 million
SK innovation, South Korea		2022	Komárom Plant 2, Hungary	9.8 GWh, option up to 16 GWh. Investment €753 million
Samsung SDI, South Korea	2.5	2017	Göd Plant 1, Hungary	2.5 GWh in 2020, expansion to 12 GWh by 2023 and 20 GWh by 2028
Samsung SDI, South Korea	7.5	2021	Göd Plant 2, Hungary	Investment €740 million. Investment incentives €108 million. In partnership with Mercedes-Benz
SK ON (SK innovation), South Korea		2028	Iváncska Plant 3, Hungary	9.8 GWh, rising to 30 GWh in 2028. Total investment by SK innovation in Hungary €1.6 billion. Investment incentives €209 million. 1900 jobs
CATL, China		2028	Debrecen, Hungary	Planned capacity: 100 GWh, investment €7.34 billion, 9000 jobs
EIT InnoEnergy, Netherlands		2025	Subotica, Serbia	The first LFP lithium-ion battery gigafactory in Europe. Construction is scheduled to begin in 2024, 8 GWh by the end of 2025, expansion to 16 GWh planned later. Based on the LFP technology developed by the Serbian company ElevenEs based in Subotica

*Source*: Based on AMS (2021b), Harrison (2021), various news reports and company press releases

**Table 6:** Selected FDI into the battery industry in EE, including the announced future investments

Company, home country	Start year	Location	Notes
SK IE Technology (SK innovation), South Korea	Plant 1: 2021, Plant 2: 2023, Plant 3: 2024, Plant 4: 2024	Dąbrowa Górnicza, Poland	Plants for separators used in electric car batteries. One thousand new jobs. Total investment USD 1.5 billion
EcoPro BM, South Korea	2024–2025	Debrecen, Hungary	Cathode material factory for electric car batteries. Investment €715 m
Enchem, South Korea	Unknown	Komárom, Hungary	Lithium salt production facility for lithium-ion batteries with annual capacity of 20,000 tons. The investment was announced in 2021
Anodox Energy, Sweden	2022	Riga, Latvia	The assembly of battery packs for EVs should start in December 2022. A second factory should follow. Investment €50 m, 300 jobs
Enchem, South Korea	2020	Biskupice Podgórne, Poland	Lithium salt production facility for lithium-ion batteries with annual capacity of 20,000 tons. The construction of the second factory in Kobierzyce announced in 2022
Capchem, China	2022	Śrem, Poland	A €50 m electrolyte production factory for 40,000 tons of electrolyte per year, 60 jobs
Daimler, Germany	2022	Jawor, Poland	Battery assembly facility, 100,000 batteries for BEVs and PHEVs per year
Northvolt, Sweden	2022	Gdańsk, Poland	Production of battery modules. An initial capacity of 5 GWh in 2022 and potential for 12 GWh
Umicore, Belgium	2022	Nysa, Poland	Cathode material factory, 400 jobs. Production capacity will grow to over 200 GWh/year to produce battery cells for 3 m EVs after 2025
SKC (SK Group), South Korea	2024	Stalowa Wola, Poland	Copper foils factory for use in EV batteries. Investment €693 m, initial capacity of 50,000 tons per year
Volkswagen (Škoda Auto), Germany	2019	Mladá Boleslav, Czechia	€130 m car battery assembly line for the VW Group’s MQB platform. The annual capacity of 380,000 MEB battery systems by the end of 2023. 250 jobs
Stellantis, France	2019	Trnava, Slovakia	The assembly of car batteries (35,922 assembled in 2020)
Dräxlmaier, Germany	2022	Timisoara, Romania	Battery systems for hybrid cars. Investment €200 million. More than 1000 jobs
Rock Tech Lithium, Germany/Canada	2029	Romania	A production plant for battery-grade lithium hydroxide. Investment €715 million. A memorandum of understanding signed in March 2022. Location unknown

*Source*: Based on various news reports and press releases

### The continuing strong location advantages for the automotive industry in EE

Fifth, EE will continue to have strong location advantages for the automotive industry in the context of the EU. These include low wages compared to WE, the geographic location close to the large and affluent west European markets, and EU membership. EE will continue to be an attractive location for potential new EV assembly plants and the production of battery cells and components. In the long run, the drive for profit of automotive companies will prevail. As long as the wages in EE continue to be significantly lower than in WE, especially in Germany, EE will be attractive for the continuing production and additional investment, including the investment in the battery industry and production of EVs (Tables 5, 6 and 7) (Pavlínek 2020). However, as already discussed, this potential can be undermined by insufficient or exhausted labor surplus despite low labor costs, as it has recently been the case in central Europe and Romania (Pavlínek 2015a; PwC 2019; Adăscăliței and Guga 2020; Guga 2019; HIPA 2020). The recent location decision of Japan’s Nidec corporation illustrates this point. In December 2021, Nidec started the construction of a factory to produce electric engines in Novi Sad, Serbia, which will employ one thousand workers. Nidec will also build a smaller factory for automotive inverters and engine control units that will create 200 jobs. Serbia has been selected for the location of these factories because of its low wages (Table 1), labor surplus (the total unemployment rate of 9.1% in 2020, down from 19.4% in 2014), and the future EU membership (Manthey 2021b; Nidec 2021; Eurostat 2021c). These factories will not be built in Poland or Hungary despite their greater recent experience in engine manufacturing (Table 8) and proximity to the market because of their higher wages (Table 1) and labor shortages that were considered more important for the location decision by Nidec. Ultimately, when the basic preconditions for automotive FDI are present, such as political stability, the absence of trade barriers with the EU, and the transportation access to the market, it is the combination of labor costs and labor availability that drives concrete location decisions in the EE automotive industry (Pavlínek 2020; HIPA 2020; Nidec 2021; Vesić and Vukša 2021).

**Table 7:** Production of EVs in EE in 2021, including the announced future investments as of 2022

Lead firm	Brands	Models	Location	Notes
Next.e.GO Mobile	e.Go	Next generation e.Go Life	Lovech, Bulgaria	A German micro-factory for up to 30,000 EVs per year should open at the beginning of 2024
Rimac	Rimac	Electric supercars	Zagreb, Croatia	The majority owner (55%) of Bugatti since 2021 in the Bugatti-Rimac JV, in which Porsche holds 45%. Low volume production
Hyundai	Hyundai	Kona EV	Nošovice, Czechia	2021 output: 22,468 BEVs (8.2% of the total vehicle output), 21, 174 PHEVs (7.7% of the total vehicle output)
VW Group	Škoda	Superb iV PHEV	Kvasiny, Czechia	2021 output: 27,919 PHEVs, including Octavia iV PHEV made in Mladá Boleslav (4.1% of the total Škoda vehicle output in Czechia)
VW Group	Škoda	Enyaq iV EV, Octavia iV PHEV	Mladá Boleslav, Czechia	2021 output: 49,701 BEVs (7.3% of the total Škoda vehicle output in Czechia). The series production of the Enyaq Coupé iV launched in February 2022
SOR	SOR	Electric buses	Libchavy, Czechia	2021 output: 60 BEVs (10.9% of the total vehicle output)
BMW	BMW	Third generation electric cars, the Neue Klasse	Debrecen, Hungary	Factory opening in 2025 to produce 150,000 EVs per year
BYD	BYD	Buses	Komárom, Hungary	The production of 200 e-buses per year since April 2017. The expansion to 1000 e-buses per year is expected to be completed in 2022
Daimler	Mercedes-Benz	EQB	Kecskemét, Hungary	Series production of the all-electric Mercedes EQB since October 2021
Suzuki	Suzuki	Vitara hybrid	Esztergom, Hungary	A “strong” hybrid should become available in 2022. Suzuki plans to offer a BEV by 2025 but it is unclear whether it will be made in Hungary
VW Group	Audi	Q3 PHEV	Györ, Hungary	The production started in December 2020 and is integrated into the existing production process of ICE cars
Next.e.GO Mobile	e.Go	e.wave X	Tetovo, North Macedonia	A German micro-factory for up to 30,000 EVs per year should open at the end of 2024
Solaris	Solaris	Electric bus	Owińska, Poland	The Polish brand owned by the Spanish CAF, 390 e-buses sold in 2021, 11.9% market share in Europe. Between 2012 and 2021, Solaris sold 1132 electric buses or 13.3% of Europe's total. In 2020, 1560 buses produced of which 44% were fully electric and one-third were hybrids
VW Group	MAN	Lion’s City E bus, hybrid bus	Starachowice, Poland	Series production of the all-electric Lion's City E-bus started in October 2020
EMP Poland	Izera	SUV and hatchback BEVs	Jaworzno, Poland	The national BEV project, majority state-owned. Assembly launch pushed from 2023 to late 2024. 15,000 jobs: 3000 in assembly, 12,000 among suppliers. Annual capacity 150,000 BEVs
Ford	Ford	Puma mild hybrid	Craiova, Romania	The Puma mild hybrid produced since 2019. Production of an all-electric version of a new LCV Puma, Ford Transit Courier panel van, and the Tourneo Courier passenger car version will start in 2024
Stellantis	Fiat	Compact EV	Kragujevac, Serbia	The start of production of small EVs planned for 2024
Kia	Kia	Ceed and XCeed PHEV	Žilina, Slovakia	In 2020, 21,000 PHEVs (8% of the total output) and 31,916 units of mild hybrid versions of the Kia Sportage and Kia Ceed (12% of total output). A BEV model planned in 2025
JLR	Land Rover	Defender, Discovery PHEVs	Nitra, Slovakia	The PHEV Defender since September 2021. A BEV Range Rover planned in 2024
Stellantis	PSA	Peugeot e-208, Batteries for all brands	Trnava, Slovakia	Peugeot e-208 BEV since 2019. 7263 BEVs in 2019 (2% of the factory output), 33,334 in 2020 (10%). 35,922 batteries assembled in 2020
VW Group	Audi, Seat, Škoda, VW, Porsche	VW e-up!, 5 SUV PHEVs (the VW Touareg, Porsche Cayenne, Porsche Cayenne Coupé, Audi Q7, Audi Q8)	Bratislava, Slovakia	In 2020, 42,275 BEVs and 28,875 PHEVs were assembled. BEVs accounted for 13.7% and PHEVs for 9.3% of the total vehicle production. Electrified vehicles accounted for 23% of the total vehicle output
Geely Volvo Cars	Volvo	Volvo EXC90, Polestar 3	Košice, Slovakia	Factory construction is planned to start in 2023 and series production in 2026. Annual capacity up to 250,000 BEVs. Investment incentives €267 m
Renault-Nissan-Mitsubishi	Renault, Smart	Twingo Electric, Clio PHEV	Novo Mesto, Slovenia	The Renault Twingo Electric is being manufactured exclusively by Revoz and accounted for one-third of its total output in 2021. The production of the Smart Forfour EQ EV ended in December 2021

*Source*: AMS (2021a), AIA (2022), various news reports and company press releases

**Table 8:** Engine and transmissions plants in EE

Country	Product	Parent company	Location
Poland	Engines	Volkswagen AG	Polkowice, Poland
	Engines	Daimler Group	Jawor, Poland
	Engines	Toyota Motor Europe	Wałbrzych, Poland
	Engines	Toyota Motor Europe	Jelcz-Laskowice, Poland
	Engines	Stellantis	Tychy, Poland
	Engines	Stellantis	Bielsko-Biała, Poland
	Transmissions	Toyota Motor Europe	Wałbrzych, Poland
Czechia	Engines	Volkswagen AG (Škoda)	Mladá Boleslav, Czechia
	Transmissions	Hyundai	Nošovice, Czechia
	Transmissions	Volkswagen AG (Škoda)	Mladá Boleslav, Czechia
	Transmissions	Volkswagen AG (Škoda)	Vrchlabí, Czechia
Romania	Engines	Renault SA	Mioveni (Pitești), Romania
	Engines	Ford Europe	Craiova, Romania
	Transmissions	Daimler	Sebeș, Romania
	Transmissions	Renault SA	Mioveni (Pitești), Romania
Hungary	Engines	Volkswagen AG	Györ, Hungary
	Engines	Stellantis	Szentgotthárd, Hungary
	Transmissions	ZF Friedrichshafen	Eger, Hungary
Slovakia	Engines	Kia (Hyundai Motor Group)	Žilina, Slovakia
	Transmissions	Getrag Ford	Kechnec, Slovakia
	Transmissions	Volkswagen AG	Bratislava, Slovakia

*Source*: Based on ACEA (2021a) and ANE (2017)

The most important limitations of these five general observations about the transition to the production of EVs are related to the highly increased geopolitical risks and volatility caused by the 2022 war in Ukraine and by the unfolding energy crisis in Europe. Energy costs multiplied in EE in 2022 compared to 2021. Combined with one of the highest dependencies of large EE vehicle producers, such as Czechia, Slovakia, and Hungary, on Russian natural gas, it may undermine one of EE’s competitive advantages in the automotive industry. For example, in September 2022, VW warned that it might relocate production away from Germany and EE to its factories in southwestern Europe or coastal areas of northern Europe because of their proximity to seaborne liquefied natural gas terminals (ANE 2022d). EE may also be impacted by the increased perceived investment risk due to its geographic proximity to Ukraine, which might negatively affect future investment decisions by TNCs in the EE automotive industry.

## Uneven effects of the transition to the production of EVs in the automotive industry

The overall trend away from the production of ICE vehicles and toward EVs will lead to the restructuring of the automotive industry in Europe (Sigal 2021; McKinsey&Company 2021). The main questions about the transition to the production of EVs in EE are about its speed and its effects, i.e., how long it will take, and how it will ultimately affect the automotive industry. However, in thinking about these effects, we need to keep in mind that the trend toward the production of EVs is only one of several important megatrends that will affect the automotive industry in EE. Other trends, such as those associated with the digitalization, robotization and automation of production (Industry 4.0), continuing investment, reinvestment, and the relocation of production, will also impact the automotive industry in EE and will likely have more important employment effects than the transition to the production of EVs (e.g., Bauer et al. 2020; Drahokoupil 2020; Szabo 2020).

The shift to the production EVs will likely disrupt employment patterns but it will disrupt them unevenly in different sectors of the EE automotive industry. The two most important sectors of the narrowly defined automotive industry employing the most workers are the production of parts and components (NACE 29.3) and the manufacture of vehicles and engines (NACE 29.1). NACE 29.3, which employed 671,590 persons in EE in 2020 (Eurostat 2022d), accounting for 78% of all automotive industry jobs, is likely to be most affected.^5^ Within NACE 29.3, suppliers of components and parts for the ICE powertrain (e.g. components and parts for engines, gear boxes, fuel, and exhaust systems) will be most affected as their products will become redundant in BEVs. For example, a combustion engine has 1018 forged components, while a comparable full electric engine has only 143 (Schwabe 2020b). The drivetrain of BEVs is less complex than in conventional vehicles and requires, for example, only half of its bearings (Davies et al. 2015). Therefore, even if the production of ICEs is replaced with the production of electric engines, it might result in significant job losses, because the production of electric engines is less labor intensive than the manufacture of ICEs (CLEPA 2021; Bauer et al. 2020). On the other hand, large segments of the supplier industry that are unrelated to ICEs will experience no or small effects (e.g., seats, wheels, structure parts, AC systems), and the new segments of the automotive industry related to the battery system will create new jobs (e.g., batteries, battery management systems, sensors). The entire battery industry, including the extraction of raw materials, manufacturing of battery cells, battery assembly, and recycling, could create up to four million jobs in the EU (Harrison 2021).

The shift to EVs might also disrupt employment patterns in NACE 29.1, which employed 160 thousand persons in EE in 2020 (Eurostat 2022d), because the assembly of BEVs is less labor intensive than the manufacturing of traditional cars. There are fewer mechanical parts and despite many new electric and electronic components and the battery, fewer workers will be needed in the final assembly. For example, to maintain the employment levels from before the transition to EVs, Volkswagen’s BEV Zwickau factory integrated some processes that used to be outsourced to external suppliers, such as stamping work for the hood, fenders, and doors. This ultimately translates into fewer jobs in the supplier sector (Gibbs 2019a). NACE 29.1 will also be affected due to the fact that the production of electric engines is less labor intensive than the manufacture of ICEs (CLEPA 2021; Bauer et al. 2020).

**Fig. 4:**
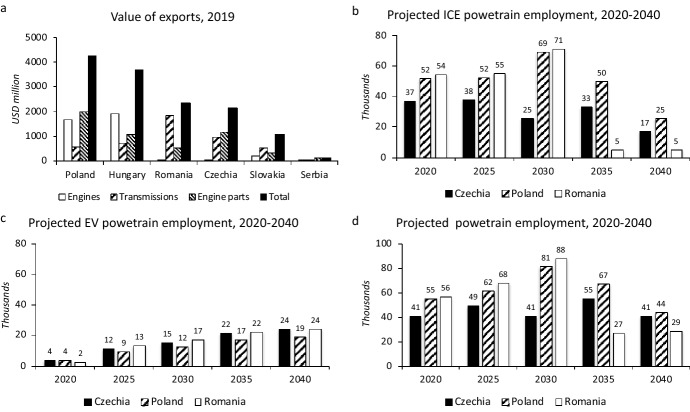
Powertrain exports and projected powertrain employment, 2020–2040. *Note*: Engines refer to ICEs, transmissions refer to transmissions for motor vehicles. *Source*: Author based on data in OEC (2022), CLEPA (2021)

These effects will be geographically uneven across EE since different EE countries are specialized to a different degree in the production of distinct automotive products and components. For example, Poland and Hungary are more dependent on exports of ICEs, engine parts and transmissions than other EE countries (Fig. 4a). The production of engines and gearboxes in Czechia and Slovakia is mainly for the large local assembly of cars and not for exports. Poland, the largest producer of engines has six engine factories (Table 8) and exported engines worth €2.8 billion in 2020 (PZPM 2022; OEC 2022). Poland, Czechia, and Hungary are also the largest exporters of engine parts from EE (Fig. 4a), making them potentially vulnerable to the decrease in the production of ICEs.

But even in the cases of ICEs, the production will not necessarily end in 2035, because some EE factories, such as Škoda Auto in Czechia and its suppliers, plan to continue to produce ICEs for foreign markets that will undergo much slower transition to electromobility, such as India, Russia, South America, and north Africa. The speed of the transformation will also differ for different segments of the supplier industry. In most cases, the change will not be abrupt, but it will be gradual and the existing engine factories might gradually transition to the production of electric engines. For example, Audi Hungária at Győr, Hungary, the largest engine factory in EE and in the world, started to produce electric engines in 2018. Out of the total number of 1,661,599 engines produced in 2020, 87,343 (5.3%) were electric axle drive units and their share will continue to increase in the future (Audi 2021) so that the factory may assemble only 271,000 ICEs in 2029 (Sigal 2022a).

Projections of changes in powertrain employment under the most likely scenario of the transition to EVs prepared for selected European countries for the 2020–2040 period by CLEPA (2021) suggest for the EE countries the maximum employment in ICE powertrain technologies around 2030, followed by a steady decline to 2040 (Fig. 4b); a steady increase in the employment in EV powertrain technologies (Fig. 4c); which, however, will not compensate for job losses in the ICE powertrain technologies. Overall, almost 50% powertrain jobs are projected to be lost in Czechia, Poland, and Romania between 2030 and 2040. Compared to 2020, the number of powertrain jobs is projected to be lower by one-fourth in these three countries in 2040 (Fig. 4d) (CLEPA 2021).

Local automotive suppliers are mostly captive Tier-three suppliers or niche suppliers in automotive GVCs/GPNs (Pavlínek 2018; Pavlínek and Žížalová 2016). As such, local firms will be in a weak position to effect any changes related to the transition to EVs in EE. Empirical research has suggested the weaking position of local firms in the EE automotive industry because of their inability to benefit from and keep up with its rapid FDI-driven growth in the 2000s and 2010s (Pavlínek 2020).

## The battery industry in eastern Europe

Attracting FDI to battery and cell manufacturing is a feasible strategy to attract the assembly of EVs, thus ensuring the future of the automotive industry in EE and offsetting job losses caused by the decreases in the production of ICEs, even though jobs in the EV battery assembly are not high value-added jobs (Szalavetz 2022) and the production of battery cells is highly automated (Schade et al. 2022). Since batteries are heavy and can account for up to one third of the total EV weight (Delanote et al. 2022), the geographic proximity of the battery assembly operations lowers transportation costs involved in transporting finished batteries to a vehicle assembly factory. There are also strong strategic reasons behind the development of the battery industry in Europe because batteries account for 30–50% of the value of BEVs (CLEPA 2022). It has been estimated that 24 new battery gigafactories with annual capacity of 25 GWh will have to be built in Europe by 2030 to meet the European battery demand (McKinsey&Company 2021). In December 2020, the EU specified its local content requirements for the European lithium battery production, which require the location of key parts of its value chain in Europe between 2024 and 2027 (e.g., cathodes, anodes, and chemicals), with the goal of achieving 100% European sourcing by 2027. The European Commission approved large subsidies for the development of the European battery industry (€3.2 billion in 2019 and €2.9 billion in 2020) (Harrison 2021). These developments will support the growth of the battery industry in EE, including an increase in high value-added jobs in battery design and testing that has already been documented in a few celebrated cases of local startups, such as InoBat in Slovakia and ElevenEs in Serbia, and also in some foreign subsidiaries (Szalavetz 2022) (Table 6). However, because the production of battery cells is very energy intensive, the future growth of the battery industry in EE is likely to be negatively affected by drastically increased energy prices and the high degree of dependence on Russian natural gas, unless alternative sources of cheap energy are found.

Lithium deposits, a crucial raw material to produce car batteries, have potentially been one of the locational advantages of EE for the development of the battery industry. Two large deposits have been discovered in EE: one in western Czechia in the Ore Mountains close to the German border, which is the largest lithium deposit in Europe (up to 3% of global lithium deposits), the other one in the Loznica region of western Serbia along the Drina River close to the Bosnia and Herzegovina border. Foreign mining TNCs, Australian European Metals Holdings (EMH) in the case of Czechia and British-Australian Rio Tinto, in the case of Serbia, were interested in mining the lithium deposits. In both cases, however, mining projects became highly politicized. In the case of Czechia, after the political outcry about foreign capital control of the lithium deposit, the government-linked energy company ČEZ purchased a majority stake in Geomet, EMH’s subsidiary, for €32 million, because it held exploration licenses for lithium deposits (Deloitte 2021). ČEZ and EHM are considering mining lithium but no decision about the mining has been made as of 2022 (HN 2019, 2021). In the case of Serbia, the government stopped the $2.4 billion mining project in January 2022 following the strong resistance of local communities and environmentalists (Randall 2022b).

The development of the battery industry in EE has so far been limited compared to WE (AMS 2021b; Williams 2021; Dunn 2022). As of August 2022, only 13.3% of completed or planned installed capacity of lithium battery gigafactory projects were in EE (Heines 2022). Within EE, the growth has so far been restricted to Hungary (5.5% of the European total) and Poland (4.9%) (Table 5). The recently announced €7.34 billion investment by Chinese CATL in a 100 GWh gigafactory in Hungary will strongly increase Hungary’s European share (Tables 5 and 6). Hungary and Poland have been more aggressive than other EE countries in attracting the battery industry perhaps because of their greater dependence on the production of ICEs (Fig. 4a) and, therefore, greater vulnerability to potential job losses related to the decrease in the production of ICEs compared to the rest of EE. In coming years, we might also expect investments in the EE battery industry from European companies, whose rapidly growing investments have so far been limited to WE (Beutnagel and Verpraet 2021; Heines 2022). It is likely that in addition to Hungary and Poland other EE countries will be targeted by FDI in the battery production.

Czechia and Slovakia, which have the largest production of cars in EE (Fig. 2a) have so far failed to attract any battery gigafactories. The Czech government has been actively attracting one of six planned VW’s gigafactories, which is supported by Škoda Auto, by offering investment incentives, including tax breaks, building of transportation infrastructure and retraining the thousands of workers (Liebreich 2021; Charvát 2022). Czechia’s EE locational advantages, such as low labor costs, the largest European lithium deposits, and the proximity of other VW factories, are being undermined by the limited state support for the transition to electromobility, the weak promotion of future technologies, and the rapidly rising energy costs since 2022 (Škoda Auto 2021a; Hampel 2021). VW is therefore also considering Poland and Hungary for its EE gigafactory, which might be a way to extract the biggest possible investment incentives from one of these three governments, a typical strategy in location decisions by automotive TNCs in the European integrated periphery (Pavlínek 2016).

In Slovakia, InoBat Auto, the Slovak startup, and the California-based Wildcat Discovery Technologies, the owner of the patented technology for car batteries, are building a €100 million 100-MWh pilot battery line and R&D center for 150 R&D workers close to the town of Trnava (Manthey 2019; Bolduc 2021). However, its 10 GWh gigafactory to produce up to 150 thousand smart batteries per year will not be built in Slovakia as originally thought but in WE (Randall 2022a). EE has also attracted a growing FDI into module and pack battery manufacturing, which bundles individual battery cells into modules and packs, as well as into material suppliers for battery production and battery recycling. A high share of these investments went to Poland (Table 7), whose government allocated €3.1 billion for investment incentives to the battery industry in 2019 (Strzałkowski 2021).

## Conclusion

This article considered the implications of the integrated periphery position of the EE automotive industry in the European GVCs/GPNs and IDL for the course of the transition to the production of EVs. It has argued that EE’s dependent position will strongly influence the course of this transition. Although there are many questions and uncertainties about this transition that have been greatly enhanced by the geopolitical and economic instability because of the war in Ukraine, several general conclusions about its nature in EE can be made. First, the extremely high dependence of the EE automotive industry on foreign ownership and control means that the course of the transition will be driven by the corporate decisions of foreign automotive TNCs. Local firms will be unable to influence the course of the transition and play a significant role. Second, while the role of the CO_2_ regulation imposed on the automotive industry at the EU level has been instrumental in triggering the transition to the production of EVs, the role of states and their policies in EE will be severely limited. It will mainly focus on attracting additional FDI from flagship foreign investors through offering various investment incentives, engaging in the ‘race to the bottom’ by competing with other states in the integrated periphery for these investments, and pursuing additional policies designed to meet the needs of flagship investors in the production of EVs. Third, the transition to the production of EVs will be slower in EE than in WE because of the continuing production of ICE vehicles, slower introduction of fully dedicated EV factories, and the greater reliance on the mixed production of EVs and ICE vehicles than in WE. While this slower transition will likely increase the employment in the EE automotive industry in the short and medium run in the 2020s, it might weaken the position of EE in the IDL of the European automotive industry in the long run by relying on the increasingly obsolete and less profitable ICE technologies and falling behind WE, because WE will move to the full-scale production of EVs in EV dedicated factories faster. Fourth, the impact of the transition to EVs will be uneven within the automotive industry. In terms of jobs, there will be a greater potential for job losses in the production of parts and components than in the vehicle assembly. The creation of new jobs in the battery industry will depend on the abilities of EE governments to attract foreign battery manufacturers. So far, however, the majority of battery gigafactories are built or planned to be built in WE, not in EE. Only Hungary and to a lesser extent Poland have so far attracted any significant investment in the battery industry. Fifth, the transition will be geographically uneven in EE. The course of the transition and its outcome in the individual EE countries will depend on their ability to attract FDI in the production EVs, the battery industry and battery components production, which will help offset potential losses related to phasing out the production of components for ICEs.

The article has identified several risks for the future competitiveness of the automotive industry in the EE integrated periphery based on the currently pursued strategies of the transition to EVs. Most importantly, the reliance on the mixed production of ICE vehicles with EVs, on the production of ICE cars for longer period than in WE, and the potential failure of some EE countries to attract the battery industry, especially battery gigafactories, might undermine their long-term competitive position in the European automotive industry. To counteract these risks, the integrated periphery will continue to rely on its enduring competitive advantage of low production costs, especially low labor costs, to continue to attract FDI in the automotive industry, which is risky because of the exhausted labor surplus in many countries. Since the transition to the production of EVs will mostly depend on foreign capital, it will continue to be dependent growth (Pavlínek 2017a), which is unlikely to improve the highly dependent position of EE countries in automotive GVCs/GPNs and the IDL in the European automotive industry.

The validity of these conclusions is generally supported by recent research (e.g., Szalavetz 2022; Delanote et al. 2022; Slačík 2022; CLEPA 2021). However, it can be strongly undermined by the effects of the increased geopolitical instability and energy crisis in Europe. EE is more vulnerable than WE due to its geographic location close to Ukraine and Russia, the high dependence on Russian energy resources, and the landlocked location of many EE countries, which makes access to alternative sources of oil and liquified gas more difficult and expensive. This might negatively affect the future investment decisions of foreign TNCs in the EE automotive industry. Therefore, given the analysis presented in this article and significant risks identified above, the best outcome of the transition to the production of EVs the EE automotive industry can hope for is to maintain its integrated peripheral position in the European automotive industry division of labor and GVCs/GPNs that has developed since the early 1990s.

## Footnotes

1 In this article, EE refers to the former state-socialist countries in central Europe, south-eastern Europe (SEE) and the Baltic states, that became EU members. It also includes non-EU member countries in SEE (Serbia, Bosnia and Herzegovina, North Macedonia, and Moldova) that have been integrated into the European automotive industry production system through investment and trade links. Russia, Ukraine, and Belarus are excluded from the analysis. Central Europe refers to Czechia, Hungary, Poland, Slovakia, and Slovenia.

2 The European automotive industry was disrupted by Russia’s invasion and war in Ukraine in February 2022 (Harrison 2022; ANE 2022c). There are 22 foreign companies producing parts and components for the automotive industry in 38 factories and employing over 60 thousand workers in Ukraine. Many are producing wire harnesses. The production of wire harnesses by Leoni, Fujijura and Nexans has been particularly affected. For example, in early March 2022, the halt of assembly of cable harnesses at two large Leoni factories in Ukraine in Stryi and Kolomyja, which employ 7000 workers, led to drastic reductions in production at Volkswagen, Audi, Porsche, BMW and Mercedes-Benz in Germany (Eddy 2022; ANE 2022b). Ukraine is also the source of 70% of the world supply of neon, which is needed to produce semiconductors as is palladium, of which about one-third is supplied by Russia. Many foreign automakers halted their Russian factories and suspended business in Russia, including Ford, Honda, Toyota, Volkswagen, Jaguar, Aston Martin, Volvo, General Motors, and Daimler Truck (ANE 2022c). Foreign automotive suppliers have also suspended their operations in Russia. For example, Magna suspended production in its six factories in Russia that employ 2500 people (Irwin 2022).

3 In 2001, compared to Germany, average personnel costs (personnel costs per employee) in the automotive industry (NACE 29) were lower by 86% in Slovakia, 85% in Czechia, 84% in Poland, 82% in Hungary, and also by 96% in Bulgaria and 95% in Romania (Eurostat 2016).

4 In 2020, the USA was the fourth largest exporter of vehicles to the EU market (after Britain, Turkey, and Japan), while China was the sixth largest. Between 2015 and 2020, the imports of American vehicles increased from 245 to 393 thousand, the imports of Chinese vehicles grew from 81 to 197 thousand. Vehicle imports from the USA increased by 10.7% in 2020 and from China by 27.5%, while they declined from all other main country origins and by 19.7% overall (ACEA 2022a).

5 The actual direct employment in the automotive industry is larger as the broadly defined automotive industry also includes firms from other industrial sectors that are involved in the automotive value chain but are not included in NACE 29, such as suppliers from the plastic industry, rubber industry, electrical equipment, and iron and steel industry.
